# Intestinal Inflammation in Chilean Infants Fed With Bovine Formula vs. Breast Milk and Its Association With Their Gut Microbiota

**DOI:** 10.3389/fcimb.2018.00190

**Published:** 2018-06-21

**Authors:** Juan C. Ossa, Dominique Yáñez, Romina Valenzuela, Pablo Gallardo, Yalda Lucero, Mauricio J. Farfán

**Affiliations:** Departamento de Pediatría y Cirugía Infantil, Facultad de Medicina, Hospital Dr. Luis Calvo Mackenna, Universidad de Chile, Santiago, Chile

**Keywords:** intestinal inflammation, bovine formula, breast milk, gut microbiota, infant cohort

## Abstract

**Introduction:** Compared to bovine formula (BF), breast milk (BM) has unique properties. In the newborn intestine, there is a homeostatic balance between the counterparts of the immune system, which allows a physiological inflammation, modulated by the gut microbiota. Many studies have attempted to understand the effect of BF vs. BM, and the changes in the gut microbiota, but few also focus on intestinal inflammation.

**Methods:** We conducted a cohort study of newborn infants during their first 3 months. In stool samples taken at 1 and 3 months (timepoints T1 and T3), we quantified calprotectin, IL-8 and α1-antitrypsin by ELISA and we evaluated the expression of *IL8* and *IL1*β genes by RT-qPCR. To determine the microbiota composition, the 16S rRNA gene was amplified and sequenced using 454 pyrosequencing. Sequences were clustered into operational taxonomic units (OTUs).

**Results:** In total 15 BM and 10 BF infants were enrolled. In the BM group, we found calprotectin and α1-antitrypsin levels were significantly elevated at T3 compared to T1; no differences were found between T1 and T3 in the BF group. A comparison between the BM and BF groups showed that calprotectin levels at T1 were lower in the BM than the BF group; this difference was not observed at T3. For IL-8 levels, we found no differences between groups. A gene expression analysis of the *IL8* and *IL1*β genes showed that infants from the BF group at T1 have a significantly increased expression of these markers compared to the BM group. Gut microbiota analyses revealed that the phylum Bacteroidetes was higher in BM than BF, whereas Firmicutes were higher in BF. A redundancy analysis and ANOVA showed BM has a community structure statistically different to BF at T1 but not at T3. Compared to BF, BM at T1 showed a higher representation of *Enterococcus, Streptococcus, Enterobacter, Lactococcus*, and *Propionibacterium*.

**Conclusions:** We found a basal state of inflammation in the infants' intestine based on inflammation markers. One month after birth, infants receiving BF exhibited higher levels of inflammation compared to BM.

## Introduction

Breast milk (BM) has been and will continue to be the ideal type of nutrition for every term or pre-term newborn. The WHO recommends exclusive breastfeeding for the first 6 months of life, with supplemental breastfeeding until 2 years old and beyond (Hoddinott et al., 2008). Compared to bovine formula milk (BF), BM contains nutrients, hormones, growth factors, immunoglobulins, cytokines and bacteria which confer protection against many diseases, such as necrotizing enterocolitis, respiratory and gastrointestinal infections, allergy, celiac disease, obesity, diabetes type I and II (Horta et al., 2007; Le Huërou-Luron et al., 2010). In the healthy newborn intestine, the counterparts of the immune system allow the mucosa to display a physiological inflammation, a result of the immune response to diet and bacteria in the intestinal lumen (Fiocchi, 1997). Gut microbiota starts to develop *in utero*, inherited from the mother, and is later influenced by the mode of delivery and the newborn feeding pattern (Bäckhed et al., 2012). Nowadays, gut microbiota plays a key role in maturation and maintenance of the immune system, food metabolism, intestinal epithelial cell homeostasis, protection against pathogens and neural development of the gut-brain axis (Hill and Artis, 2010; Lathrop et al., 2011). The shift in the composition of a healthy microbiota to an unhealthy one is called dysbiosis. Currently, many enteric and non-enteric diseases have been associated with dysbiosis of the gut microbiota (Arrieta et al., 2014).

Most studies have attempted to understand the effect of BF or BM on the gut microbiota composition showing that BF feeding is associated with microbiota with lower abundance of Bacteroides, and higher Clostridia compared to BM-fed infants. Regarding Bifidobacteria, there is a controversy as to whether they are lower in number and frequency in BF than BM. Also, BF-fed infants exhibit higher counts of Enterobacteriaceae than BM-fed infants (Guaraldi and Salvatori, 2012; Fan et al., 2014). Other studies have shown the effect of diet on the intestinal cell homeostasis in healthy neonates by either analyzing gene expression from exfoliated epithelial cells or protein levels in stools. Although several inflammatory markers as well as inflammatory gene expression have been evaluated in stool or serum in infant, few studies have addressed diet in the newborn looking at intestinal inflammation and its relation to changes in gut microbiota composition (Chapkin et al., 2010; Savino et al., 2010). Considering the above, using a non-invasive technique based on stool analysis, we conducted a 3-month cohort study of newborn infants who are either in the exclusive BM or BF to determine inflammatory markers in stool and gut microbiota composition.

## Methods

### Study design

We conducted a 3-month cohort study following 2 groups of newborn infants who are fed exclusively either with BM or BF. All infants were recruited from the maternity ward of Hospital Luis Tisné in Santiago, Chile. In order to associate the effect of diet with inflammation marker levels and the microbiota composition over the 3-month period, a collection of stool samples were taken at 1 (timepoint T1) and 3 (timepoint T3) months of life, within a range of ±5 days. The timepoints were chosen due to the limited knowledge about the intestinal inflammation and its association with gut microbiota under 6 months.

### Patients

*Inclusion Criteria*. We enrolled infants born at term (38–42 weeks of gestation), vaginally delivered, and described healthy at the time of discharge from the hospital. For enrollment, all the infants had to be receiving BM or BF exclusively. The BF group also included infants who were in BM and receiving formula supplements ≥20% of the volume ingested that day. *Exclusion Criteria*. We excluded from the study infants or mothers who during the study period received antibiotics, probiotics, steroidal or non-steroidal anti-inflammatory drugs 1 month prior to enrollment. Also, we excluded from the study mothers hospitalized other than for delivery, for surgical intervention, serious infection, or with any sign or symptom of infection or gastrointestinal disease (diarrhea, vomiting, fever).

### Clinical assessment

During recruitment, a complete clinical interview was done to take the history regarding pregnancy, delivery mode, birth weight, frequency and quantity of feeding, stool frequency, family composition, number of siblings, history of allergy, gastrointestinal and other systemic disorders in the family, family income and household environment (number of rooms, water supply and pets in the home).

### Sample collection

A stool sample was obtained from the infants during the endpoints described above. Samples were collected during a clinical visit to our center, the Hospital Luis Calvo Mackenna, Santiago, Chile. In case that the infant has no stool during the visit, a home kit for the parents was given to take the sample and store it in a sterile container to be transported to our center within the following 6 h. Stool samples were divided into at least 4 aliquots and stored at −80°C.

### Ethics

This study was done in accordance with the recommendations of the Declaration of Helsinki. The study protocol was approved by the ethical committees of the Servicio de Salud Metropolitano Oriente and Hospital Luis Tisné. Written informed consent was obtained from all parents on behalf of their infants.

### Inflammatory protein markers determination

ELISA commercial kits for stool samples were used for the analysis of calprotectin (IDK® calprotectin ELISA, Immunodiagnostik, Germany) and α1-antitrypsin (IDK® α1-antitrypsin ELISA, Immunodiagnostik, Germany). Samples were processed as directed by the manufacturer. For IL-8, we determined the concentration of these markers by ELISA as previously described (Harrington et al., 2005).

### Gene expression assay

RNA was isolated from the stool sample using the Stool Total RNA Purification Kit (Norgen Biotek) according to the manufacturer's instructions. Then, cDNA was synthesized using the First Strand cDNA Synthesis Kit (ThermoFisher Scientific) and RT-qPCR was carried out with the TaqMan Gene Expression Assay (ThermoFisher Scientific) and specific TaqMan probes for *IL8, IL1*β, and *GADPH* genes as previously described (Bennett et al., 2009; Chapkin et al., 2010). The *GADPH* gene was used as a housekeeping gene to normalize the expression of *IL8* and *IL1*β. Changes in cycle threshold (ΔCT) values for each gene were obtained at T1 and T3. The mean of the ΔCT of the BM group was used as a reference for the fold expression changes in the BF group.

### Pyrosequencing and operational taxonomic unit (OTU) assignment

Total DNA was extracted from stool samples using the QIAamp Fast DNA Stool Mini Kit (Qiagen) and stored at −20°C until PCR amplification. The 16S rRNA gene was amplified in a two-step process. First, the 16S rRNA gene was amplified using the primers GM3 and 1492R, and then a nested PCR was performed using the GM3-PS forward primer and a different 907-PS reverse primer for each sample in a 7-cycle reaction as described (Gallardo et al., 2017). Amplicons were purified and the concentration of the purified product was determined. Equimolar mixtures of the amplicons (10–12 samples each) were shipped to Macrogen Inc. (Seoul, Korea) for pyrosequencing. Pyrosequencing of each mix was done through 454 GS-FLX using a 1/8 plate. Sequence trimming and OTU assignment were performed by Macrogen using CD-HIT-DUP and QIIME (Caporaso et al., 2010) according to their standard protocol [cutoff of 97% of sequence identity at species level for OTU assignment and using the 11th version of RDP-16s rDNA database as reference (http://rdp.cme.msu.edu/index.jsp)]. The sequence data reported in this study have been deposited in the European Nucleotide Archive (ENA) database, under accession number PRJEB25846.

### Statistics

Gene expression and protein results are expressed as means ± standard error of the mean (SEM). Comparison of results between multiple groups was performed using one-way analysis of variance (ANOVA) and the Tukey-Kramer multiple comparisons test for results between the different experimental groups. For the OTU comparison analysis we used a non-parametric bootstrapping method, the Mann-Whitney u test. Differences with a *P* < 0.05 were considered statistically significant. Analyses were performed using Prism6 (GraphPad, San Diego, California, USA). Redundancy analysis (RDA) of OTU composition was done using the “vegan” package of version 3.4.2 of the R software, as described by Gallardo et al. (2017). The abundance of each taxa was normalized by the total diversity per sample prior to any group comparison. For the taxonomic analysis, the abundance of each taxa was normalized by the total diversity per sample prior to any group comparison.

## Results

### Patient demographic

During the study, 25 patients were recruited, 15 in the BM and 10 in the BF group. There were no differences between gender or birth weight and lengths. The number of stools/day was lower in the BF group (2.5 vs. 4.4), but this difference was not statistically significant. All the patients had the same average number of relatives in the home. Regarding pets and allergies, all the families had pets and 50% of the patients in both groups had a history of first-grade atopy (atopic dermatitis or asthma or allergic rhinitis or food allergy). Finally, family income was similar in both groups. Table 1 summarizes these findings.

**Table 1:** Patient demographics of BM and BF groups.

**Patient characteristics**	**BM group (*n* = 15)**	**BF group (*n* = 10)**	***p*-value**
Sex (F/M)	7/8	6/4	ns
Birth weight avg. (gr)	3,457	3,400	ns
Birth length avg. (cm)	51	50.2	ns
Stools per day avg.	4.4	2.5	ns
Family members avg. (n)	5	5	ns
History 1st degree atopy	50%	50%	ns
Pets at home	60%	80%	ns

*ns, not significant*.

### BF-fed infants showed higher intestinal inflammation than BM-fed infants

**Figure 1:**
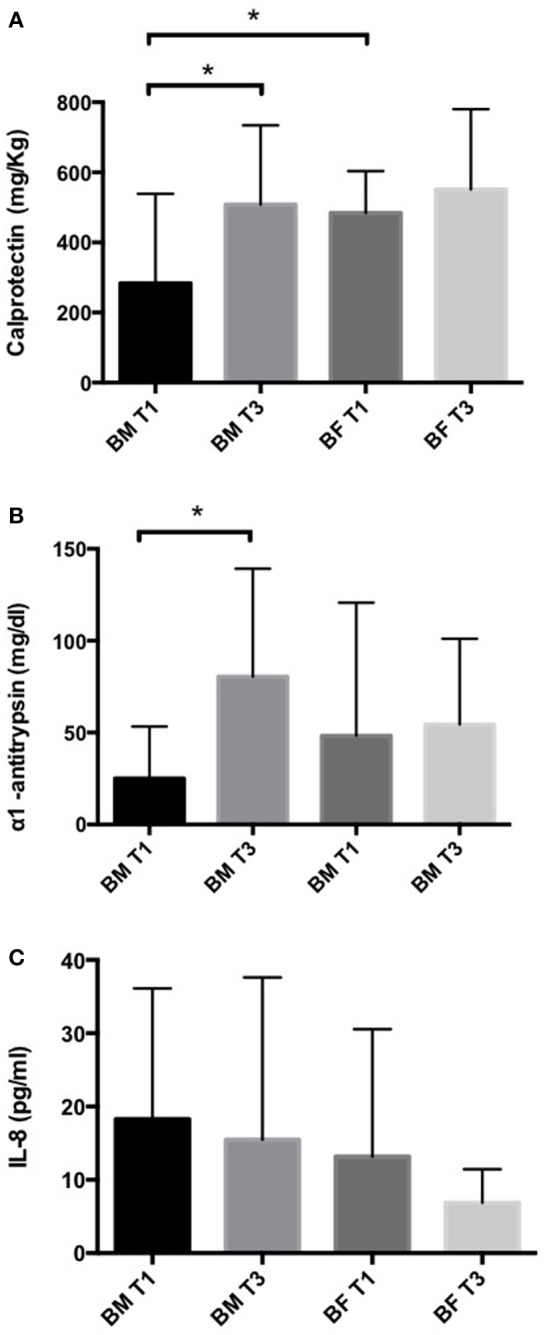
Inflammatory markers in stool samples of infants fed with BM or BF. Concentration of calprotectin **(A)**, α1-antitrypsin **(B)** and IL-8 **(C)** in stool samples from BM and BF groups were determined by ELISA. ^*^p < 0.05.

We quantified the levels of inflammatory markers calprotectin, IL-8 and α1-antitrypsin in stool samples by ELISA. In the BM group, we found calprotectin and α1-antitrypsin levels were significantly higher at T3 than at T1. No differences were found between T1 and T3 in the BF group (Figures 1A,B). A comparison of the BM and BF groups showed that calprotectin levels at T1 were lower in the BM group; this difference was not observed at T3 (Figure 1A). For IL-8 levels, we found no difference between groups (Figure 1C).

**Figure 2:**
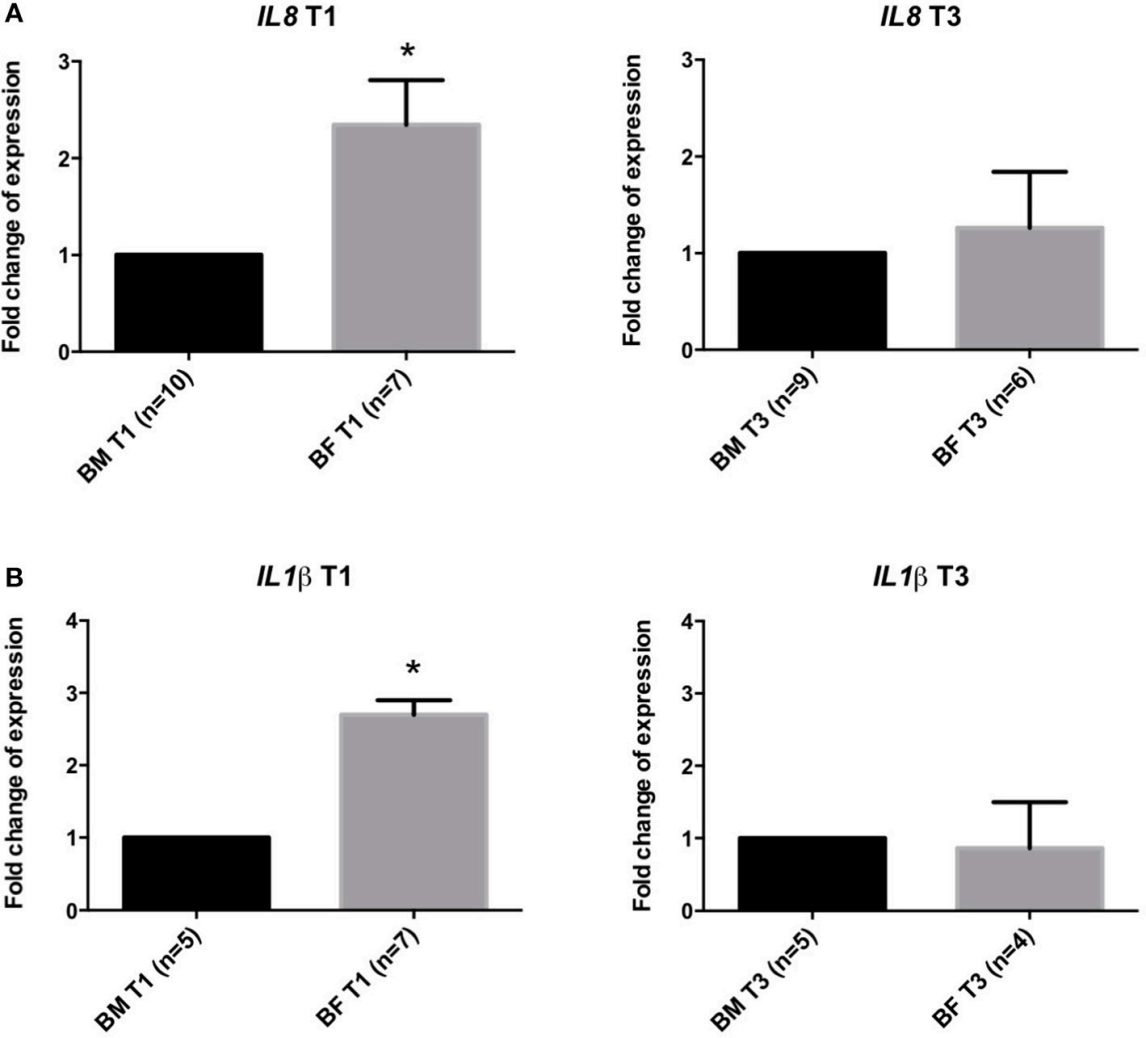
Gene expression analysis in stool samples of infants fed with BM or BF. Gene expression at timepoints T1 and T3 of *IL8* **(A,B)** and *IL1*β genes in stool samples from BF group compared to BM group. Changes in cycle threshold (ΔCT) values for each gene, normalized to GAPDH gene, were obtained at T1 and T3. The mean of the ΔCT of BM group was used as a control for the ΔCT expression in the BF group compared to the BM group. ^*^p < 0.05.

Gene expression analysis of *IL8* and *IL1*β genes showed that infants from the BF group have a significantly increased expression of these markers (2.3 ± 0.45 and 2.3 ± 0.4-folds, respectively) at T1 compared to the BM group. At T3, no difference was found in the expression of these genes (Figure 2).

### BF-fed infants harbor a different intestinal microbiota than BM-fed infants

**Figure 3:**
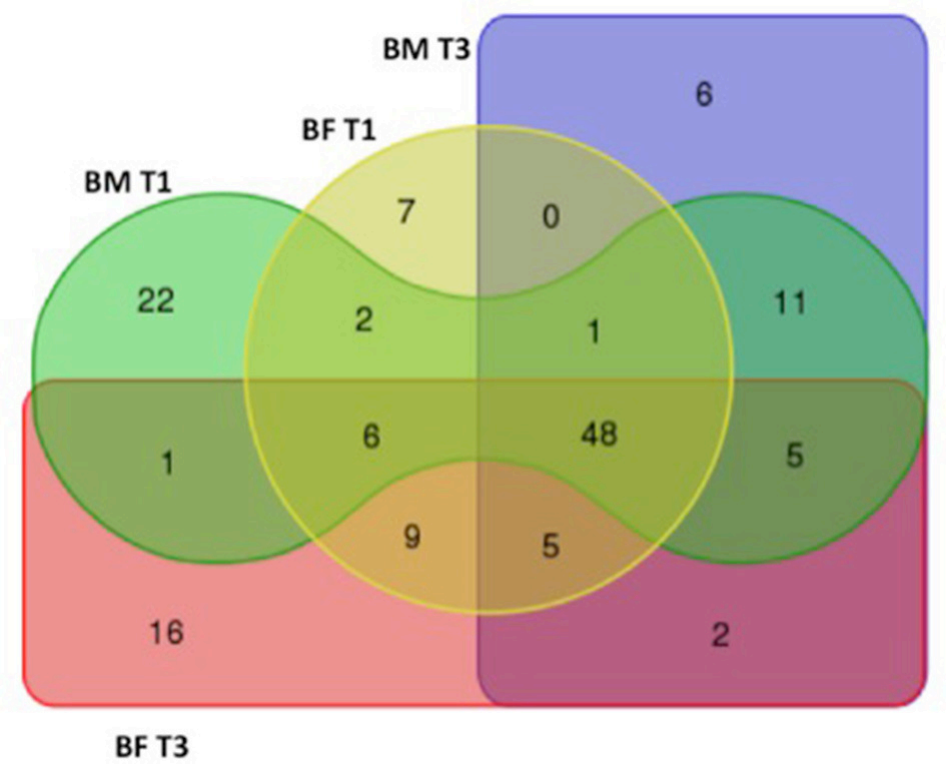
Distribution of the OTUs among groups. Venn diagram showing the distribution of the 140 OTUs found at BM T1 (green), BM T3 (purple), BF T1 (yellow), and BF T3 (red) timepoints.

**Figure 4:**
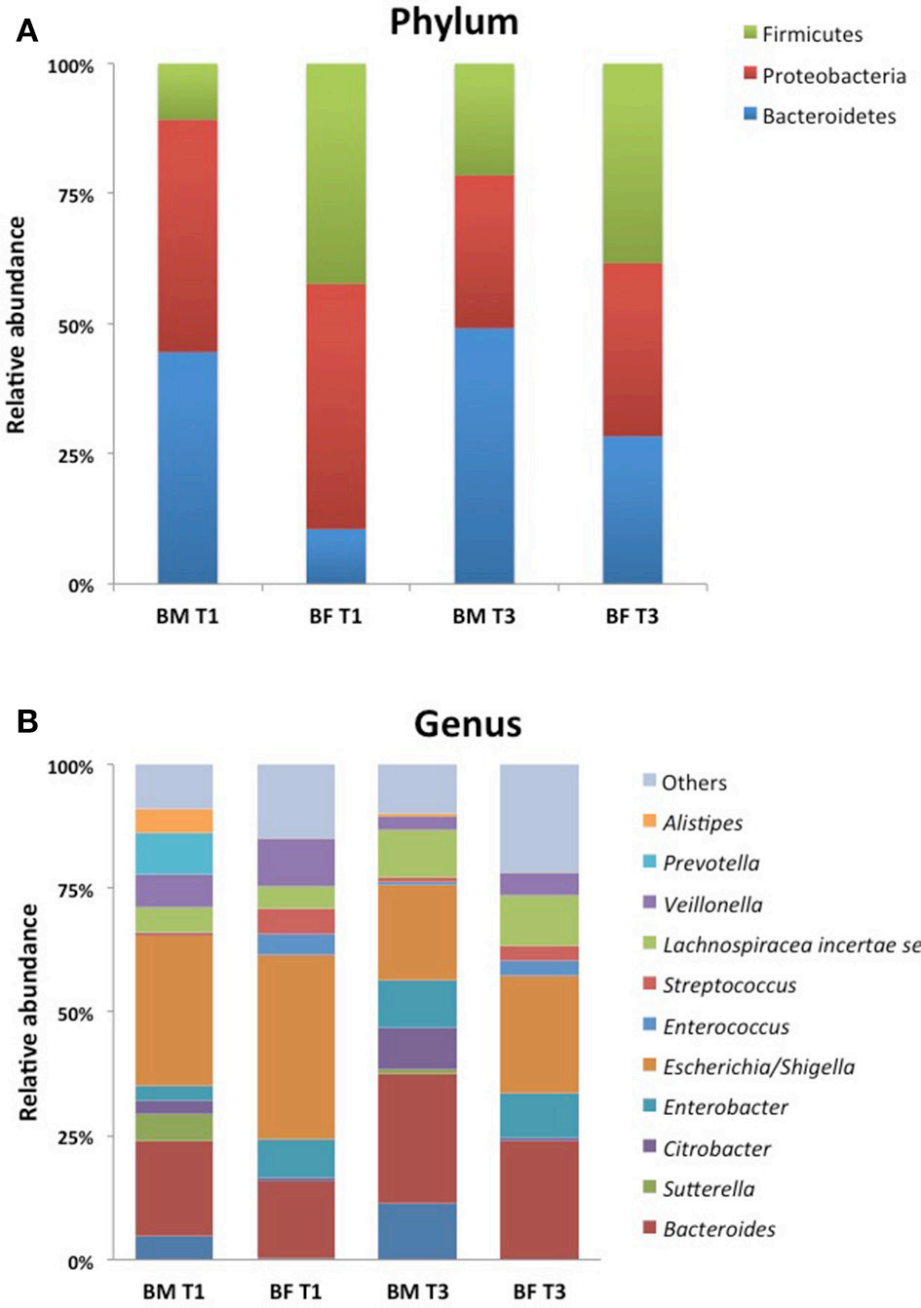
Community profile at different taxonomic levels. Relative abundance of taxa at Phylum **(A)** and Genus **(B)** level of the 10 most abundant taxa. Each color represents a different taxonomic unit. Less representative taxa were grouped as “other”.

The taxonomic analysis showed a total number of 140 OTUs, 28 exclusively present in the BM group and 23 in the BF group. Shared OTUs among groups was only 2 at T1, but at T3 the number of shared OTUs increased to 48. When comparing the microbiota at T1, BM-fed newborns had 22 exclusive OTUs compared to 6 in the BF group (Figure 3). At phylum level, we found the BM group had a lower Firmicutes proportion (20 and 23%) than the BF group (38 and 42%) at T1 and T3. By contrast, a higher Bacteroides presence at T1 and T3 was found in the BF group (38 and 47%) than in the BM group (16 and 25%), respectively. The Firmicutes/Bacteroides ratio was lower in the BM group than the BF group at T1 (0.5 vs. 2.4) and at T3 (0.5 vs. 1.7). The proportions of Proteobacteria and Actinobacteria were similar among all groups. At genus level, the most abundant genera in all groups were *Escherichia/Shigella* and *Bacteroides*, followed by *Parabacteroides, Lechnospiracea*, and *Veillonela* (Figure 4).

A comparison of OTUs at genus level showed that the BF group had a higher representation of *Enterococcus* (*p* = 0.001), *Streptococcus* (*p* = 0.001), *Enterobacter* (*p* = 0.01), *Lactococcus* (*p* = 0.03) and *Propionibacterium* (*p* = 0.04**)** than the BM group at T1. At T3, the BM group had a higher representation of *Sutterella* (*p* = 0.04) and *Parabacteroides* (*p* = 0.04), whereas BF-fed infants had higher number of *Streptococus* (*p* = 0.01).

**Figure 5:**
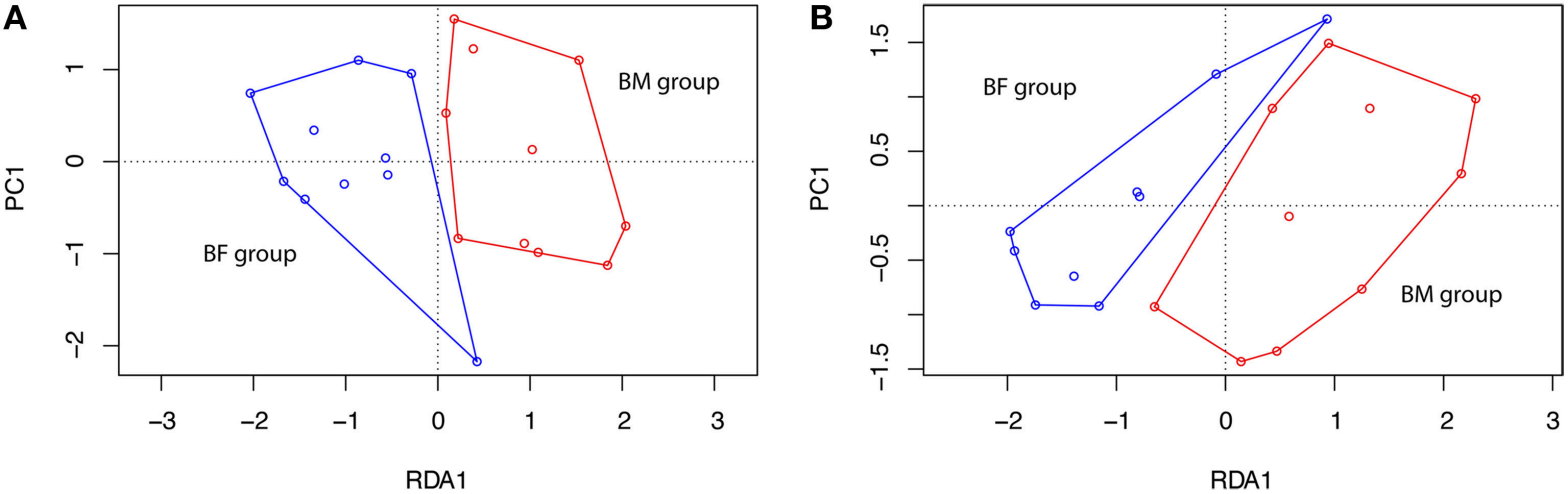
Redundancy analysis at timepoints T1 and T3. A redundancy analysis (RDA) was conducted using sample classification as the explanatory matrix and relative OTU diversity as the response matrix at **(A)** T1, 1 month and **(B)** T3, 3 months. Data was normalized with a double square root transformation. Sample grouping and axis significance were analyzed by ANOVA (RDA T1 *p* = 0.003, RDA T3 *p* = 0.238).

A RDA analysis of OTU composition showed a significant difference between BM and BF groups at T1 (Figure 5A), but not at T3 (Figure 5B).

## Discussion

Breastfeeding confers important benefits on the infant and protection from many diseases, most of them associated with changes in the intestinal tract environment. Few studies have endeavored to address the effect of diet and intestinal inflammation on the newborn. Here, we have shown in a cohort study that BF-fed infants have a higher intestinal inflammation defined by an increased concentration of calprotectin and α1-antitrypsin in stool samples taken 1 month after birth (T1) compared to an infant fed exclusively with BM. Even though these differences were not observed 2 months later (T3), our data support the role of BM in the low-grade inflammation compared to BF-fed infants 1 month after birth. Calprotectin and α1-antitrypsin are markers that specifically express protein loss and inflammation as seen in several gastrointestinal disorders, such as allergies and inflammatory bowel diseases (Poullis et al., 2002; Saarinen et al., 2002). Previous reports have compared the calprotectin levels in stools in healthy infants, BM vs., BF (median age 51 days old). Interestingly, the stool calprotectin level was higher in the BM group than in the BF group, suggesting a possible degree of local inflammation in the intestine in the BM infants (Savino et al., 2010). In another study, no differences were found in the stool calprotectin level between BM and BF newborns at 3 months old (Rosti et al., 2011). Similar to our findings but with a different approach, Kainonen et al., using a cohort of infants fed with BM and BF at high risk for the development of allergies, compared the levels of INF-γ, TNF-α, and IL-2 (proinflammatory), IL-5 and IL-4 (allergy) and IL-10 and TGF-β2 (anti-inflammatory). The BM group showed significantly lower proinflammatory markers in serum compared to the BF group, and the TGF-β2 levels in the BF group were significantly lower than in the BM group. These findings lasted up to 1 year, despite supplementation with solid food in both groups. Finally, they suggested BM had an immunomodulatory role “protecting” against inflammation (Kainonen et al., 2012). Possible mechanisms in breast milk dampening inflammation might involve its components, including immunoglobulins, cytokines such as IL-10, defensing, macrophage colony stimulating factors secreted by mammary epithelial cells and TGFβ produced by leukocytes present in the milk (Hennet and Borsig, 2016). In light of our results, the contribution of calprotectin and α1-antitrypsin in the gut homeostasis in infants merits further investigation.

We also quantified IL-8 in stools, but no significant differences were found between the groups. Although IL-8 is a pivotal molecule that orchestrates tissue inflammation, its role in intestinal inflammation in healthy children is not well characterized. In order to clarify the involvement of this cytokine, we decided to evaluate the expression of the *IL8* gene and another pro-inflammatory gene, *IL-1*β, in stool samples. We found a significantly increased expression of both genes in the BF group compared to the BM group at T1 but not at T3, suggesting that both genes might have a role in BM in ameliorating inflammation in the first month.

Microbiota has emerged as an important environmental factor in the inflammation of several gut diseases in children (Lu and Ni, 2015). In healthy infants, microbiota might play an important role in gut homeostasis. Our data suggest that the gut microbiota of the BM group clearly differs from the BF infants at T1 (Figure 5). BF harbors more Firmicutes and fewer Bacteroidetes, exhibiting a higher Firmicutes/Bacteroidetes (F/B) ratio than the BM group at T1 and T3, which is in line with previous data in healthy newborns (Mariat et al., 2009). A similar increase in Firmicutes has been seen in babies initially born by cesarean section (Hill et al., 2017). Interestingly, obesity is associated with an abundance of Firmicutes and a depletion of Bacteroidetes, where the interrelation of short-chain fatty acids fermented by bacteria plus the lipopolysaccharide from gram negative bacteria will induce inflammation and obesity (Chakraborti, 2015; Koliada et al., 2017). At genus level, we found that the BF group had a significantly higher amount of *Enterococcus, Enterobacter* and also *Streptococcus* than the BM group at T1, and all of these had been shown to be responsible for sepsis in early neonates as well in animal models receiving BF (Nakayama et al., 2003; Simonsen et al., 2014). These observations might be associated with a difference in calprotectin levels found in the BM group at this timepoint. Interestingly, in the BM group the calprotectin and α1-antitrypsin levels were higher at T3 than at T1, and these genera were found to be more abundant at T3 than at T1, suggesting that species belonging to these genera might be linked to gut inflammation. Our data support the idea that diet induces microbial changes that can induce inflammation and that BM reduces the inflammation burden, modulating the microbiota and thus maintaining intestinal homeostasis.

Our study has limitations. The number of patients included in the study was small, a situation that might be explained by the number of mothers who currently breastfeed their child exclusively in the first months of life. In the gut microbiota analysis, we could not identify any Bifidobacterium known to be present in newborn samples, despite other studies having found differences in their abundance between BF and BM (Penders et al., 2006; Hascoët et al., 2011). These results may possibly be attributed to the sequencing platform used (454 pyrosequencing). New sequencing platforms will make it possible to overcome this issue in future projects. Another limitation was the number of cytokines and genes evaluated. Although calprotectin, IL-8 and α1-antitrypsin are key markers of intestinal inflammation, there are several markers that could be explored. The development of new multiplex analyte platforms validated for stool samples will provide a broader picture of the molecules involved in intestinal homeostasis in infants.

In conclusion, using non-invasive methods in stools we found a basal state of inflammation baseline in the infant's intestine based on inflammation markers. At 1 month after birth, infants receiving BF exhibited higher levels of inflammation than BM in terms of changes in the microbiota composition. These results might signify that BM has a protective role in ameliorating inflammation, modulating the intestinal microbiota during the first months of life.

## Author contributions

JO participated in the study design, data acquisition, data interpretation, manuscript writing and final approval of the manuscript. DY participated in the sample collection and analysis. RV participated in the cohort enrollment and sample collection. PG participated in the microbiota data analysis and manuscript writing. YL participated in the study design and data interpretation. MF participated in the study design, data acquisition, data interpretation, manuscript writing and final approval of the manuscript.

### Conflict of interest statement

The authors declare that the research was conducted in the absence of any commercial or financial relationships that could be construed as a potential conflict of interest.
